# Placental pathology predicts infantile physical development during first 18 months in Japanese population: Hamamatsu birth cohort for mothers and children (HBC Study)

**DOI:** 10.1371/journal.pone.0194988

**Published:** 2018-04-10

**Authors:** Chizuko Yaguchi, Hiroaki Itoh, Kenji J. Tsuchiya, Naomi Furuta-Isomura, Yoshimasa Horikoshi, Masako Matsumoto, Ferdous U. Jeenat, Muramatsu-Kato Keiko, Yukiko Kohmura-Kobatashi, Naoaki Tamura, Kazuhiro Sugihara, Naohiro Kanayama

**Affiliations:** 1 Department of Obstetrics and Gynecology, Hamamatsu University School of Medicine, Hamamatsu, Japan; 2 Research Center for Child Mental Development, Hamamatsu University School of Medicine, Hamamatsu, Japan; Johns Hopkins University, UNITED STATES

## Abstract

The present study aimed to investigate the relationship between placental pathological findings and physiological development during the neonate and infantile periods. Study participants were 258 infants from singleton pregnancies enrolled in the Hamamatsu Birth Cohort for Mothers and Children (HBC Study) whose placentas were stored in our pathological division. They were followed up from birth to 18 months of age. Physiological development (body weight and the ponderal index [PI]) was assessed at 0, 1, 4, 6, 10, 14, and 18 months. Placental blocks were prepared by random sampling and eleven pathological findings were assessed, as follows: ‘Accelerated villous maturation’, ‘Decidual vasculopathy’, ‘Thrombosis or Intramural fibrin deposition’, ‘Avascular villi’, ‘Delayed villous maturation’, ‘Maternal inflammatory response’, ‘Fetal inflammatory response’, ‘Villitis of unknown etiology (VUE)’, ‘Deciduitis’, ‘Maternal vascular malperfusion’, and ‘Fetal vascular malperfusion’. Mixed model analysis with the use of the *xtmixed* command by the generic statistical software, Stata version 13.1., identified ‘Accelerated villous maturation’ and ‘Maternal vascular malperfusion’ as significant predictors of a lower body weight and ‘Deciduitis’ as a significant predictor of a small PI, throughout the first 18 months of life. In conclusion, the present study is the first to demonstrate that some pathological findings of the placenta are associated with changes in infantile physical development during the initial 18 months of life in the Japanese population.

## Introduction

The placenta is the largest fetal organ that links the mother to the fetus and supports most parts of organogenesis through the transport of nutrients, gases, and hormone synthesis [1, 2]. The placenta adapts to the maternal environment by changing its structure as well as function, thereby contributing to the maintenance of fetal development throughout the pregnant period. A recent programing hypothesis revealed that epigenetic changes during the early critical periods are closely associated with health and diseases in later life [3–6]. Environmental factors *in utero* are critical to the appropriate development as well as function of the entire organ system in later life.

Among heterogeneous placental components, the villous structure plays pivotal roles in the supply of nutrients and oxygen from the maternal circulation, thereby enabling proper fetal development and functions [7, 8]. A villous structure is also the main contributor to the expression of various types of bioactive substances that maintain pregnancy, including human chorionic gonadotropin, progesterone, estradiol, estriol, leptin, and resistin [9].

Placental malfunction may result in various types of fetal deterioration, such as a non-reassuring fetal status and fetal growth restrictions [10]. Placental pathology has been used in the assessment of placental conditions including malfunction and is referred to as the “memory of a pregnancy” [10]. Placental pathology permits clinicians to study the intrauterine environment of the fetus and some of the fetal responses to maternal diseases. Because placental pathology represents not only pathophysiological changes, but also physiological placental adaptations to various environmental factors, such as infection, malcirculation, chronic hypoxia on the maternal and fetal sides, and maternal hyperglycemia [10, 11]. We recently demonstrated that changes in specific lipid profiles in the villi were responsible for pathologically abnormal placental findings using a two-dimensional imaging system based on a matrix-assisted laser desorption/ionization (MALDI)-based mass spectrometer [12]. We also showed that assisted reproductive technology affected the morphology of the placental basal plate [13].

Increasing evidence suggests that physiological as well as pathophysiological changes in the placenta, including those with adaptations to the surrounding conditions, are connected not only to fetal well-being, but also health and diseases after birth [1, 14]. Khalief et al. reported that placental size negatively correlated with mental health in children and adolescents [15]. A large number of studies have examined the relationship between placental histology and the outcomes of newborns in cases of severe intrauterine infections, preterm labor, and fetal hypoxia [16–19]. However, to the best of our knowledge, the relationship between placental pathology and infantile growth after birth has not yet been investigated in the Japanese population.

In the present study, we hypothesized that the characteristics of placental pathology are related to body weight and/or composition during the infantile period in the Japanese population. The Hamamatsu Birth Cohort for Mothers and Children (HBC Study) was designed to elucidate the early developmental trajectories of children living in the community in Japan [20, 21]. In the present study, we performed a comprehensive analysis to identify links between infant physical development and pathological placental findings using 258 whole placentas from singleton pregnancies, which were stored in our pathological division, among 1,258 pregnant women who were enrolled in the HBC study.

## Methods

### Subjects

This study was conducted as part of an ongoing cohort study (the HBC Study), which has been described by Tsuchiya et. al. [20, 21]. We consecutively contacted all pregnant women (n = 1,258) who were expected to give birth at our two research sites, the Hamamatsu University Hospital and Kato Maternity Clinic, both situated in Hamamatsu city, and who gave birth between 20 December 2007 and 31 October 2011. We previously established that the enrolled parturients were representative of Japanese parturients in terms of age, socioeconomic status, parity, and the birthweight and gestational age of the child [20, 21]. Among 1,258 subjects, we initially analyzed 261 whole placentas from singleton pregnancies because the parents had agreed to store their whole placentas in our pathological division. However, we further excluded three placentas from the analysis: one infant had died, another had a confirmed diagnosis of Down’s syndrome with severe congenital heart disease, and the parents of the remaining infant refused this study after delivery. The remaining 258 (98.9%) placentas were analyzed.

All participating parturients were given a complete description of the study, and provided written informed consent to participate. They were followed from entry into the study during mid-pregnancy to 18 months after childbirth.

### Preparation of placental tissue blocks

After weighing and checking the gross morphology, whole placentas were stored in our pathological division after being vacuum-sealed in plastic packages with 10% formaldehyde (0.1 M sodium citrate buffer, pH 7.4). Seven paraffin blocks were systematically obtained from each placenta for the pathological examination by systematic random sampling, as previously described [12, 13]. In brief, linear parallel slices of placental tissue, 5mm width, were made approximately every 3cm interval perpendicular to the greatest dimension of placental axis. Then, all of the linear slices were vertically cut into small pieces every 3cm interval. Total seven blocks per a placenta were made from randomly selected seven pieces of placental parenchymal tissue thus obtained. Each block was made vertically from the maternal side to the fetal side. The two rolls of extraplacental membranes, per a placenta, were together embedded in a block for making a single section. Each block was cut into 3-μm-thick sections and followed by hematoxylin and eosin (HE) staining. Then, total eight sections (seven sections from placental parenchyma and one section from extraplacental membrane) were analyzed per a placenta.

### Pathological examination

**Fig 1:**
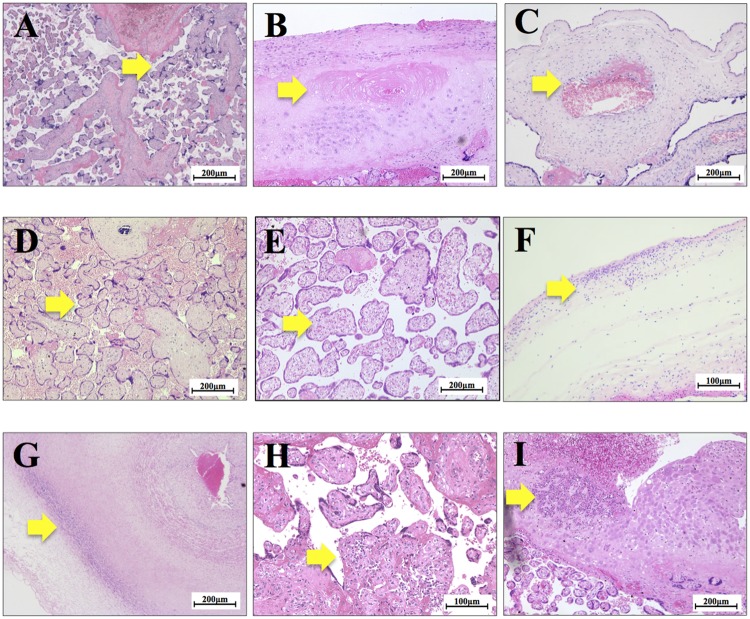
Representative pathological findings by HE staining in placentas. (A) ‘Accelerated villous maturation’; the yellow arrow indicates increases in the numbers of placental villi with the focal formation of tight adherent villous clusters with syncytial knots. (B) ‘Decidual vasculopathy’; the yellow arrow indicates the thrombus in decidual vessels. (C) ‘Thrombosis or Intramural fibrin deposition’; the yellow arrow indicates the fibrin cushion in the walls of stem villous vessels. (D) ‘Avascular villi’: the yellow arrow indicates a villi with hyalinized stroma which is devoid of vessels. (E) ‘Delayed villous maturation’; the yellow arrow indicates increases in the size of distal villi, increases in the numbers of stromal cells, and interstitial fluid uniformly distributed throughout the villous stroma. (F) ‘Maternal inflammatory response’; the yellow arrow indicates the infiltration of neutrophils in to the chorionic plate. (G) ‘Fetal inflammatory response’; the yellow arrow indicates the infiltration of neutrophils in to the umbilical vessel. (H) ‘VUE’; the yellow arrow indicates lymphohistiocytic inflammation predominantly in the stroma of terminal villi. (I) ‘Deciduitis’; the yellow arrow indicates the infiltration of lymphocytes and macrophages.

The pathological findings of placentas were classified into eleven categories with modifications from our recent study [12], in consideration of the current Amsterdam Placental Workshop Group Consensus Statement [22], i.e. ‘Accelerated villous maturation’; Fig 1A, ‘Decidual vasculopathy’; Fig 1B, ‘Thrombosis or Intramural fibrin deposition’; Fig 1C, ‘Avascular villi’; Fig 1D, ‘Delayed villous maturation; Fig 1E, ‘Maternal inflammatory response’; Fig 1F, ‘Fetal inflammatory response’; Fig 1G, ‘Villitis of unknown etiology (VUE)’; Fig 1H, ‘Deciduitis’; Fig 1I, ‘Maternal vascular malperfusion’. and ‘Fetal vascular malperfusion’

**Fig 2:**
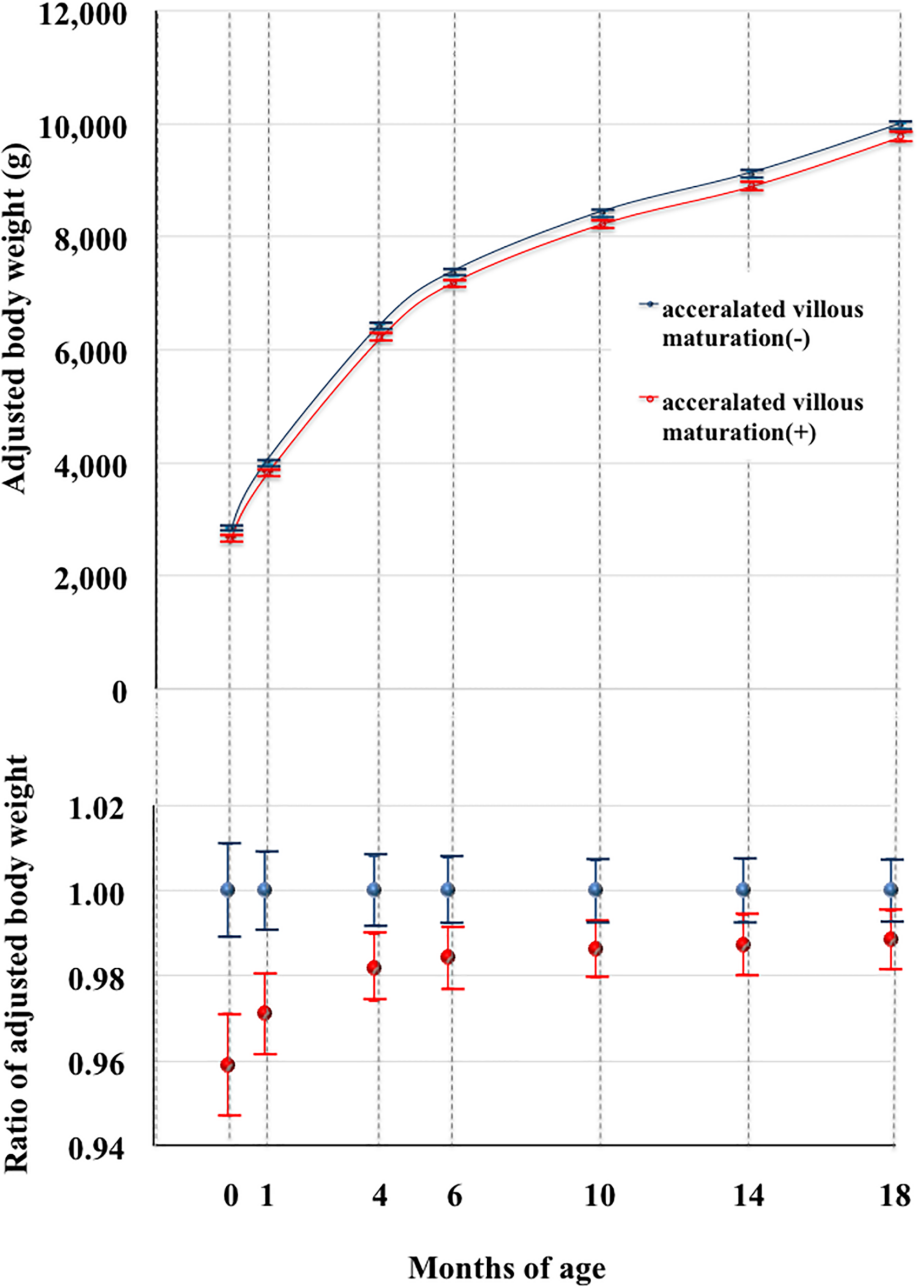
Relationship between body weight and ‘Accelerated villous maturation’ by a mixed model analysis. Upper and panels indicate the marginal mean and SD values of body weights and the relative ratio of the marginal mean of body weights. Red and blue dots indicate infants with and without ‘Accelerated villous maturation’, respectively. Error bars indicate standard deviations. ‘Accelerated villous maturation’ was a significant predictor of a light body weight in the first 18 months of life by mixed model analysis (p<0.001).

**Fig 3:**
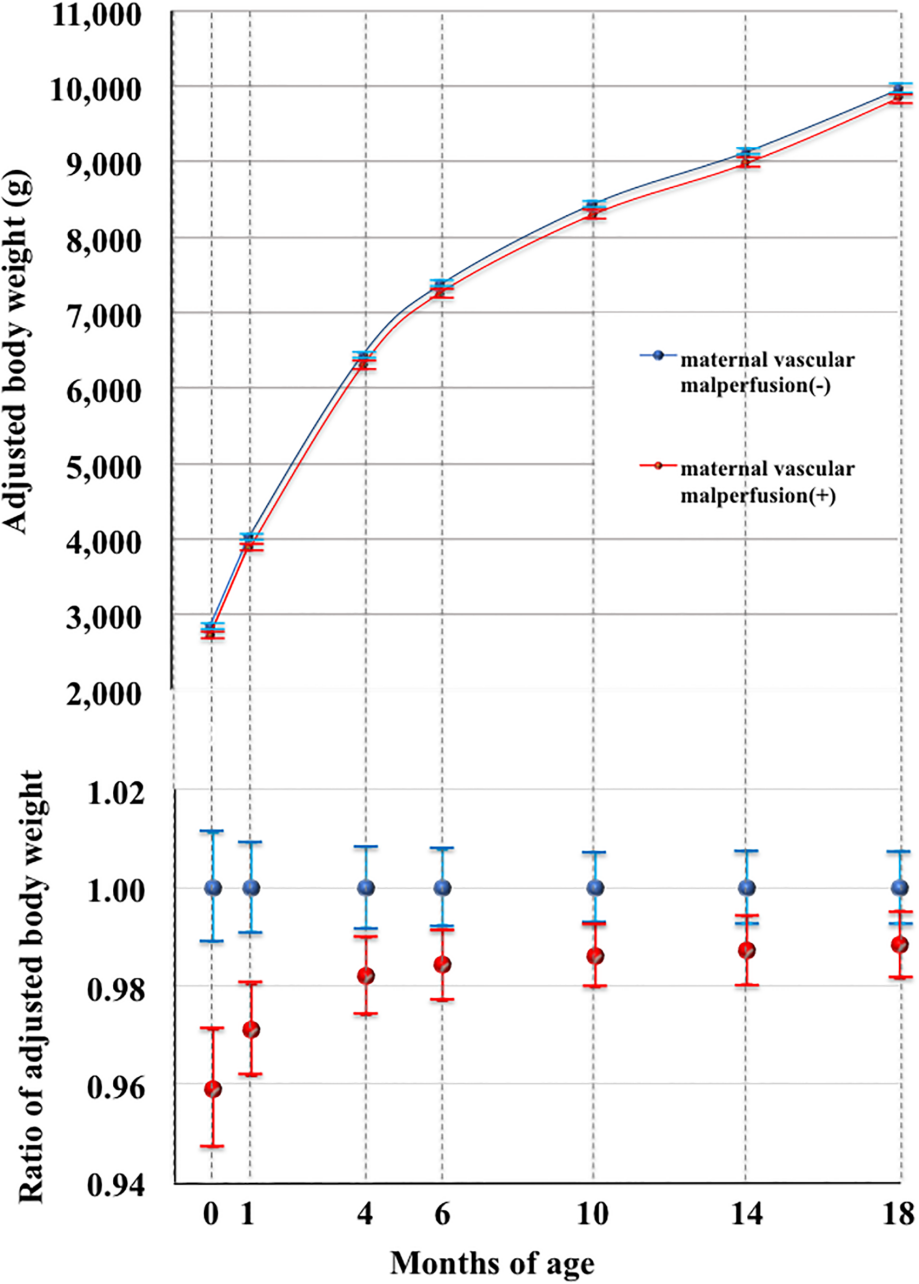
Relationship between body weight and ‘Maternal vascular malperfusion’ by a mixed model analysis. Upper and lower panels indicate the marginal mean and SD values of body weights and the relative ratio of the marginal mean of body weights. Red and blue dots indicate infants with and without ‘Maternal vascular malperfusion’, respectively. Error bars indicate standard deviations. ‘Maternal vascular malperfusion’ was a significant predictor of a light body weight in the first 18 months of life by mixed model analysis (p = 0.020).

**Fig 4:**
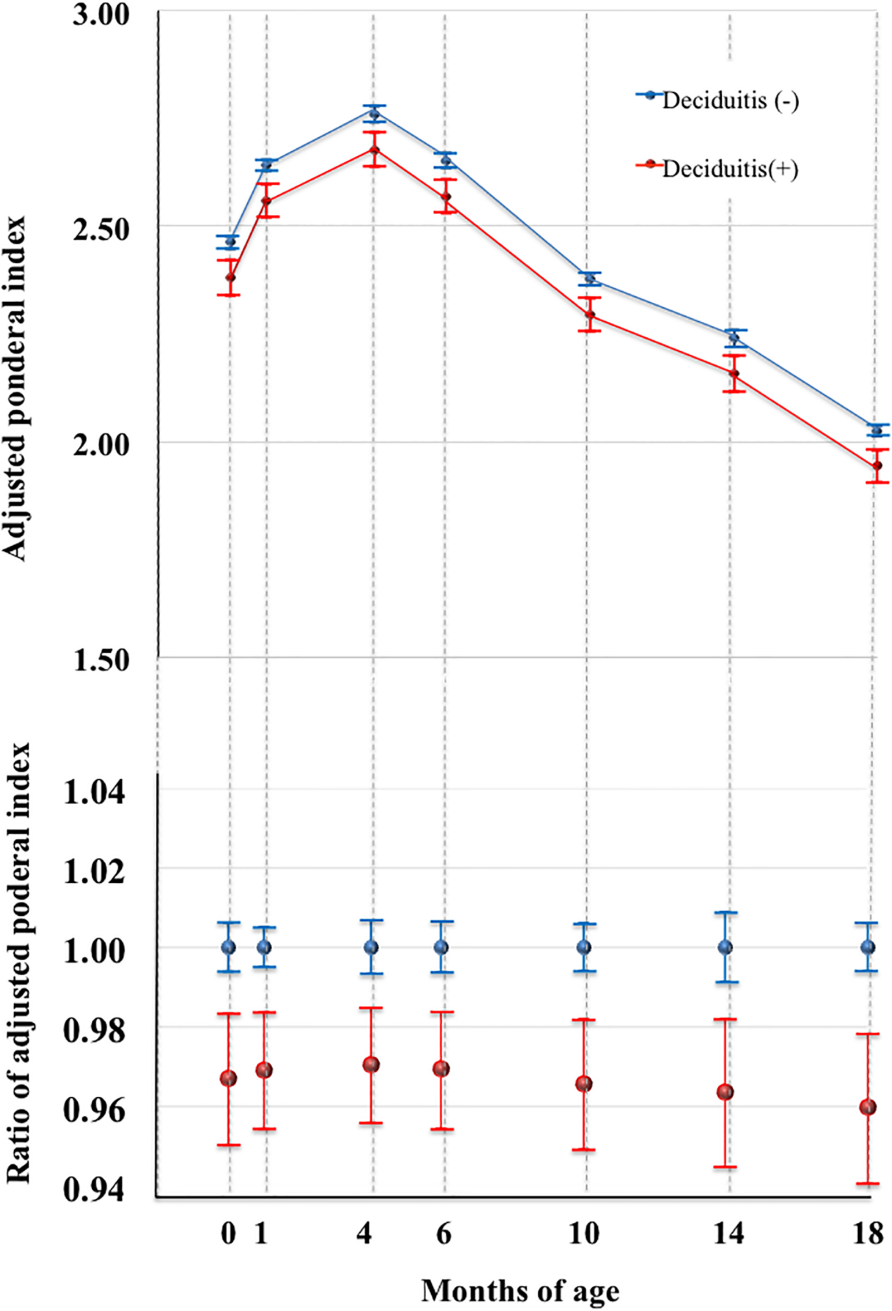
Relationship between PI and ‘Deciduitis’ by a mixed model analysis. Upper and lower panels indicate the marginal mean and SD values of the ponderal index (PI) and relative ratio of the marginal mean of PI. Red and blue dots indicate infants with and without ‘Deciduitis’. Error bars indicate standard deviations. ‘Deciduitis’ was a significant predictor of a small composition during the first 18 months of life by mixed model analysis (p = 0.035).

1) ‘Accelerated villous maturation’ was diagnosed as increased numbers of placental villi with the focal formation of tight adherent villous clusters [10, 11, 22–24] typically with syncytial knots, increased peri-villous fibrin, and the distal villous hypoplasia of small terminal villi [25] (Fig 1A), 2) ‘Decidual vasculopathy’ was diagnosed as vascular lesions including the fibrinoid necrosis of decidual vessels or atherosis found at the basal plate. [10, 22–24, 26] (Fig 1B), 3) ‘Thrombosis or intramural fibrin deposition’ was diagnosed as localized, protuberant mural lesions composed of proliferating fibroblasts intermixed with fibrin and erythrocytes in the walls of large placental vessels according to the description of Desa [10, 11, 22–24, 27] (Fig 1C), 4) ‘Avascular villi’ was diagnosed as a total loss of villous capillaries and bland hyaline fibrosis in an older lesion. [22, 24, 28] (Fig 1D), 5) ‘Delayed villous maturation’ was diagnosed as a monotonous villous population with reduced numbers of vasculosyncytial membranes, increases in the size of distal villi, increased numbers of stromal cells, and interstitial fluid uniformly distributed throughout the villous stroma [10, 11, 22–24, 29] (Fig 1E), 6) ‘Maternal inflammatory response’ was diagnosed by the infiltration of neutrophils into the connective tissues of the chorionic plate and/or amnion basement membrane in the fetal surface of the placenta [10, 11, 22–24, 30] (Fig 1F), 7) ‘Fetal inflammatory response’ was diagnosed by the infiltration of neutrophils into umbilical vessels or chorionic plate vessels. [10, 11, 22, 24, 26] (Fig 1G), 8) ‘VUE’ was diagnosed by lymphohistiocytic inflammation predominantly localized to the villous stroma of terminal villi, despite no clinical symptoms of apparent infection in mothers or infants [10, 11, 22–24, 31] (Fig 1H), and 9) ‘Deciduitis’ was enrolled as one of the findings of ‘others’, following the criteria of Amsterdam Placental Workshop Group Consensus Statement [22], which was diagnosed by the chronic infiltration of increased numbers of lymphocytes, macrophages, and plasma cells into the decidual layer, often accompanied by chronic villitis or decidual necrosis [10, 26] (Fig 1I). ‘Maternal vascular malperfusion’ and ‘Fetal vascular malperfusion’ were diagnosed according to Amsterdam Placental Workshop Group Statement [22, 24].

In the present study, each of the eleven pathological findings was assessed as positive or negative by a majority decision of independent and blind surveys by three researchers, i.e. Drs. Chizuko Yaguchi, Naomi Furuta-Isomura, and Masako Matsumoto.

### Assessment of infantile growth

During the follow-up, participating mothers and their infants were asked to visit our laboratory at the ages of 1, 4, 6, 10, 14, and 18 months in order to measure their height and weight according to the cohort schedule [20, 21]. The ponderal index (PI) was calculated as follows: Body weight ×100 / (Height) ^3^ (g/cm^3^). Some participants did not show at the examinations, which led to the lack of information for no more than four examinations out of seven in total. There were 0, 43, 54, 150, 59, 173, and 66 missing observations at 0, 1, 4, 6, 10, 14, and 18 months, respectively. In addition, some participants were late in visiting our laboratory, e.g. one month later than the expected date of the examination. In that case, we corrected measurements along with age in days using linear regression models where weight or PI were regressed onto age in days.

Information on the demographic characteristics of mothers was collected during the pregnancy of enrolled parturients and included the age of the mother, parity, smoking, and pre-pregnant height and weight [20, 21]. Perinatal variables were collected from medical records, including gestational age, birthweight, and sex [20, 21].

### Statistical analysis

Body weight and PI at 0, 1, 4, 6, 10, 14, and 18 months were set as dependent variables, and pathological findings as the independent variable. Continuous variables were reported as the mean ± SD. In comparisons of body weight or PI between two groups in the initial assessment (positive vs negative for each individual pathological finding), we performed the Student’s *t*-test or Mann-Whitney U test where appropriate. Since multiple simple comparisons were performed repeatedly for two parameters, i.e. body weight and PI, at 7 points, i.e. 0, 1, 4, 6, 10, 14, and 18 months, significance was kept conservative and, thus, set at 0.003 (0.05/[2x7]).

In order to assess the longitudinal trajectories of weight, height, and PI, we adopted a method of mixed modeling with the use of the *xtmixed* command provided by the generic statistical software, Stata version 13.1. Mixed modeling has the strength of analyzing longitudinal patterns of development in association with a fixed effect of placental pathology that occurred long before the anthropological measurements took place after birth. Furthermore, mixed modeling allows us to use observations with missing values, i.e., we incorporated all available data into the analysis even if some data had missing values in the repeated measurement of weight and PI. A growth curve model, i.e. mixed modeling with a random intercept and random slope, was built for weight, height, and PI, respectively, during 1 to 18 months of age, adjusted for age in months. Since child growth standards in terms of weight and height are typically expressed with no lower than a third order function [32], the adjustment of age in months was conducted as a linear combination of linear, quadratic, and cubic terms, which were all shown to be significant. A covariance matrix structure was set as “unstructured” because no pairs of variables were assumed to be statistically independent. Since four pairs of siblings were included, the clustering option was considered. We then incorporated all available covariates deemed to be potential confounders into the above analysis. Covariates included in the analysis were maternal parity, pre-pregnancy maternal body weight, pre-pregnancy maternal body mass index (BMI), maternal body weight just before delivery, maternal BMI just before delivery, maternal height, maternal age, maternal smoking, gestational weeks, placental weight, placental area, infantile gender, infantile months of age, household income, maternal education, maternal smoking after birth, and postpartum depression three months after childbirth which may be related to placental pathological findings, infant body weight, and PI. Mixed model analyses for longitudinal data were conducted between 0 and 18 months in order to identify differential effects on weight development and PI between the presence and absence of several pathological findings. However, if any of these covariates were shown to be non-significant (i.e. p>0.05), they were omitted from the analysis in order for degrees of freedom to be minimized. We subsequently entered all available indices of placental pathology into the above analysis, shown as the final results. The marginal means and SDs of weight and PI, resulting from the significant effects of placental pathology findings, if confirmed, were calculated while averaging the effects of all covariates. All p-values were two-sided and significance was set at 0.05 for the mixed model analysis.

### Ethical considerations

The Ethics Committee of the Hamamatsu University School of Medicine approved all procedures (No. 20–82, 21–114, 22–29, 24–67, 24–237, 25–143, 25–283, E14-062, 17–037). Written informed consent was obtained from the participating parturients during pregnancy after a full explanation of the study.

## Results and discussion

Tables 1 and 2 summarized the perinatal backgrounds of the participating mothers and infants. Tables 3 and 4 summarized placental measurements and numbers of pathological findings detected, respectively, in the placenta enrolled. The mean body weight and PI of the two groups compared (negative vs positive groups for each individual pathological finding) were summarized in Tables A-K in S1 File.

**Table 1:** Perinatal backgrounds of subjects (1).

N = 258	Mean or n	SD	Range
Maternal age (yr.)	32.5	5.23	(17–44)
Maternal body weight (kg)	53.7	11.29	(37.5–115)
Maternal BMI (non-pregnant) (kg/m^2^)	21.6	4.06	(15.9–40.4)
Maternal BMI (kg/m^2^)	25.7	4.03	(17.0–42.0)
Body weight gain (kg)	10.2	5.18	(-10.5–26.1)
Birth weight (g)	2792.9	552.5	(1126–4286)
Gestational age at birth	38.4	1.93	(29–42)
Umbilical arterial pH	7.27	0.07	(6.83–7.49)
Household income (million JPY/ year)	6	2.82	(1.00–23.00)
Maternal education (Year)	13.8	2.06	(9–23)
Maternal smoking after birth	n = 15 (5.8%)		
Postpartum depression (3month after child birth)	n = 29 (11.2%)		

**Table 2:** Perinatal backgrounds of subjects (2).

N = 258		Numbers
Gender of newborns	Male	134
	Female	124
Parity	0	142
	1	76
	2+	40
Term birth		223
Preterm birth		35
Mode of delivery	Vaginal	87
	Vacuum Extraction	22
	Cesarean Section	149

**Table 3:** Placental measurement of the subjects in 258 placentas investigated.

N = 258	Mean	SD	Range
Placental weight (g)	530.91	119.66	(230–930)
Placental area (cm^2^)	235.34	56.18	(230–930)
Cord length (cm)	53.18	11.6	(27–90)
Birth weight/Placental weight ratio (%)	5.357	0.877	(2.4–7.8)

**Table 4:** Detection of pathological findings in 258 placentas investigated.

Pathological findings	n	% *
Accelerated villous maturation	67	25.97
Decidual vasculopathy	92	35.66
Thrombosis or Intramural fibrin deposition	76	29.46
Avascular villi	26	10.08
Delayed villous maturation	50	19.38
Maternal inflammatory response	103	39.92
Fetal inflammatory response	55	21.32
VUE	15	5.81
Deciduitis	14	5.43
Maternal vascular malperfusion	120	46.51
Fetal vascular malperfusion	86	33.33

*The total percent was greater than 100 because of cases with multiple findings

‘Accelerated villous maturation’ (Fig 1A) was a significant predictor of a light body weight throughout the first 18 months of life by a mixed model analysis (p<0.001, Fig 2, Table 5) after adjusting for potential confounders, as described in the Methods. Mean body weight was significantly lower than in those without ‘Accelerated villous maturation’ using a simple statistical comparison (p<0.001, Table A in S1 File) during the first 4 months. ‘Accelerated villous maturation’ was not a predictor of a low PI by the mixed model analysis (Table 6). Collectively, these results strongly support the concept that the placental pathological finding of “Accelerated villous maturation” predicts a light body weight, but not a small body composition, at least during the first 18 months of life.

**Table 5:** Mixed model analysis of infantile body weight during the first 18 months of life.

	covariate effect	Std. Err.	p	95% Conf. Interval	
**Accelerated villous maturation**	**-190.4**	**51.83**	**<0.001**	**-291.96**	**-88.76**
Decidual vasculopathy	-8.19	55.33	0.882	-116.64	100.25
Thrombosis or Intramural fibrin deposition	9.82	57.84	0.865	-103.54	123.19
Avascular villi	-15.34	82.61	0.853	-177.26	146.58
Delayed villous maturation	51.72	61.24	0.398	-68.31	171.76
Maternal inflammatory response	33.76	67.4	0.616	-98.34	165.86
Fetal inflammatory response	-6.8	86.13	0.937	-175.6	162.01
VUE	-2.12	85.61	0.98	-169.91	165.67
Deciduitis	49.04	78.56	0.532	-104.93	203.01
**Maternal vascular malperfusion**	**-116.3**	**50.15**	**0.02**	**-214.59**	**-18.02**
Fetal vascular malperfusion	18.13	52.53	0.73	-84.83	121.09

Significance was set at a p value of 0.05 as described in the Methods.

**Table 6:** Mixed model analysis of infantile PI during the first 18 months of life.

	covariate effect	Std. Err.	p	95% Conf. Interval	
Accelerated villous maturation	-0.043	0.024	-1.79	0.073	-0.09
Decidual vasculopathy	0.012	0.021	0.56	0.575	-0.029
Thrombosis or Intramural fibrin deposition	0.006	0.023	0.24	0.808	-0.04
Avascular villi	-0.01	0.032	-0.31	0.758	-0.731
Delayed villous maturation	-0.001	0.024	-0.05	0.962	-0.048
Maternal inflammatory response	0.009	0.025	0.37	0.709	-0.04
Fetal inflammatory response	0.02	0.029	0.69	0.488	-0.037
VUE	0.048	0.035	1.37	0.17	-0.02
**Deciduitis**	**-0.082**	**0.039**	**0.035**	**-1.584**	**0.059**
Maternal vascular malperfusion	-0.006	0.02	-0.32	0.751	-0.454
Fetal vascular malperfusion	0.001	0.021	0.07	0.945	-0.04

Significance was set at a p value of 0.05 as described in the Methods.

‘Accelerated villous maturation’ is one of the abnormal villous branching patterns and suggested to be associated with hypoxic conditions *in utero* [12, 33, 34]. A large number of studies have suggested the strong suppressive effects of fetal hypoxia on physical development after birth in cases of severe fetal growth restriction. The present study is the first to demonstrate that possible hypoxic conditions *in utero* represented by ‘Accelerated villous maturation’ may be linked to a light body weight after birth, in the Japanese population.

Interestingly, the recent concept of ‘Maternal vascular malperfusion’, by Amsterdam Placental Workshop Group Consensus Statement [22], was identified as a significant predictor of a light body weight throughout the first 18 months of life by a mixed model analysis (p = 0.020, Fig 3, Table 5). Maternal vascular malperfusion’ was not a predictor of a low PI by the mixed model analysis (Table 6).

Therefore both ‘Accelerated villous maturation’ and ‘Maternal vascular malperfusion’, are predictors of a light body weight, but not a small composition, during first 18 months (Figs 2 and 3, Tables 5 and 6). Although two pathological findings independently predicts small body weight, it is plausible that potential low supply of maternal blood into placental intervillous space represented by ‘Maternal vascular malperfusion’ might be causatively associated with suspected fetal hypoxic condition represented by ‘Accelerated villous maturation’. Therefore, it was speculated that both two placental conditions, represented by specific two placental pathological findings, together might contribute, at least partly, to the programing of a light body weight during early infantile period. Physiological as well as epigenetic researches are necessary to prove this speculation.

In contrast, ‘Deciduitis’ (Fig 1I) was identified as a significant predictor of a low PI during the first 18 months of life by mixed model analysis (p = 0.035, Fig 4, Table 6), although there was no significant difference in the PI of infants with or without ‘Deciduitis’ (Table I in S1 File).

Interestingly, ‘Deciduitis’ was not a predictor of a heavy or light body weight (Table 5). Therefore, ‘Deciduitis’ may be associated with a small body composition, but not a light body weight.

‘Deciduitis’ (Fig 1I) indicates chronic inflammation mainly by macrophages and lymphocytes [35]. By contrast, ‘Maternal inflammatory response’ (Fig 1F) as well as ‘Fetal inflammatory response’ (Fig 1G) is diagnosed by the infiltration of neutrophils, indicating rather acute phase of inflammation in comparison with ‘Deciduitis’ of macrophages and lymphocytes [35, 36]. Previous studies demonstrated that these inflammatory findings were characteristic in small newborns and/or infants in a restricted population of preterm deliveries [37]. However, to the best of our knowledge, there have been no studies on the Japanese population. Unlike preterm deliveries, the body weights of infants from placentas with ‘Maternal inflammatory response’ or ‘Fetal inflammatory response’ were significantly heavier than those without by a simple comparison during first six months (p<0.001, Tables F and G in S1 File), although they were not predictors of a heavy body weight by the mixed model analysis (Table 5). We currently cannot fully explain this discrepancy. Since most of our cases of ‘Maternal inflammatory response’ or ‘Fetal inflammatory response’ were term deliveries, we speculate that the maturity of fetal organs at the time of the exposure to inflammatory cytokines may have been involved in this distinct discrepancy in the pattern of physical development after birth. The PI of infants from placentas with ‘Maternal inflammatory response’ or ‘Fetal inflammatory response’ was similar to those without these findings (Tables F and G in S1 File). They were not predictors of a high PI by the mixed model analysis (Table 6).

Thus, rather chronic phase of inflammation by ‘Deciduitis’ significantly predicts infantile small composition (p = 0.039, Table 6, Fig 4). By contrast, rather acute phase of inflammation by ‘Maternal inflammatory response’ or ‘Fetal inflammatory response’ was associated with a heavy body weight during early 6 months (p<0.001, Tables F and G in File), although they were not predictors (Table 5). Collectively, these results prompted us to speculate that chronic and acute inflammatory reactions *in utero* may distinctively affect changes in infantile body weight as well as body composition.

‘Decidual vasculopathy’, ‘Thrombosis or Intramural fibrin deposition’, ‘Avascular villi’, ‘Delayed villous maturation, ‘Maternal inflammatory response’, ‘Fetal inflammatory response’, ‘VUE’, and ‘Fetal vascular malperfusion’ were not predictors for body weight or PI during first 18 months (Tables 5 and 6).

The present study demonstrated that the characteristics of intrauterine circumstances, suggested by placental pathology, were associated with infantile physiological growth patterns. The possible hypoxic condition represented by ‘Accelerated villous maturation’ as well as ‘Maternal vascular malperfusion’ was a significant predictor of a light body weight (p<0.001 and p = 0.020, respectively; Table 5, Figs 2 and 3). The chronic inflammatory condition suggested by ‘Deciduitis’ was a significant predictor of small PI (p = 0.035, Table 6, Fig 4). The former two connections were prominent soon after birth (Figs 2 and 3), whereas the latter was consistent at least during the first 18 months (Fig 4). Therefore, it is plausible that chronic intrauterine exposure to hypoxia and inflammatory cytokines might program a light infantile body weight and small composition, respectively, in an exquisitely distinctive manner.

The limitations of this study were as follow) We did not measure inflammatory cytokine levels in cord blood, and 2) The pH of umbilical arteries at delivery, representing acute changes during parturition, did not always coincide with the presence of the chronic hypoxic findings of ‘Accelerated villous maturation’ and/or ‘Fetal vascular malperfusion’ (data not shown). Despite these limitations, the present results support the concept that some placental pathological observations predict infantile physiological growth patterns.

The concept of ‘preemptive medicine’ was recently proposed as a new preventive strategy for the current prevalence of non-communicable diseases (NCDs), i.e. the identification of high-risk populations and early interventions during a latent period before the onset of apparent clinical symptoms [5, 38, 39]. The recent developmental origins of health and disease (DOHaD) concept highlights the promising future contribution of perinatal, neonatal, and infantile care to the establishment of ‘preemptive medicine’ against the rapid spread of adult and senile NCDs [6, 40]. One important concept of preemptive medicine is identifying high-risk individuals in early life [5, 38, 39]. Considerable efforts have been made over the past few decades to establish effective biomarkers for use in clinical practice that may identify individuals at high risk of developing NCDs. The application of ‘omics’ technologies has generated hundreds to thousands of biomarker candidates. However, only a very small number of these have been translated into clinical care [41, 42]. The present study showed that some placental pathological findings are associated with changes in infantile body weight as well as body composition in the Japanese population, suggesting that placental pathological findings are applicable as a type of biomarker for predicting physical growth and/or body composition after birth.

In conclusion, the present study is the first to demonstrate that some pathological findings of the placenta are associated with changes in infantile physical development during the initial 18 months of life in the Japanese population.

## Supporting information

S1 File Tables A-K. Means and SDs of weight and the ponderal index (PI), stratified by the negative/positive of each placental pathological finding.
(DOCX)

## References

[1] Tarrade A, Panchenko P, Junien C, Gabory A. Placental contribution to nutritional programming of health and diseases: epigenetics and sexual dimorphism. J Exp Biol. 2015;218(Pt 1):50–8. Epub 2015/01/09. doi: 10.1242/jeb.110320 . 25568451 10.1242/jeb.110320

[2] Burton GJ, Jauniaux E. What is the placenta? Am J Obstet Gynecol. 2015;213(4 Suppl):S6.e1, S6–8. doi: 10.1016/j.ajog.2015.07.050 . 26428504 10.1016/j.ajog.2015.07.050

[3] Gluckman PD, Hanson MA. Developmental Origins of Health and Disease. Cambridge: Cambridge University Press; 2006.

[4] Hanson MA, Gluckman PD. Early developmental conditioning of later health and disease: physiology or pathophysiology? Physiol Rev. 2014;94(4):1027–76. doi: 10.1152/physrev.00029.2013 . 25287859 10.1152/physrev.00029.2013 PMC4187033

[5] Itoh H, Kanayama N. Nutritional conditions in early life and risk of non-communicable diseases (NCDs); the perspective of preemptive medicine in perinatal care. Hypertens Res Preg. 2015;3:1–12.

[6] Itoh H, Kanayama N. Developmental Origins of Health and Diseases (DOHaD); Perspective toward Preemptive Medicine. Singapore: Springer Nature; 2017.

[7] Larsen W. Human embryology. Philadelphia: Churchill livingstone; 2001.

[8] Robbins JR, Bakardjiev AI. Pathogens and the placental fortress. Curr Opin Microbiol. 2012;15(1):36–43. doi: 10.1016/j.mib.2011.11.006 . 22169833 10.1016/j.mib.2011.11.006 PMC3265690

[9] Sagawa N, Yura S, Itoh H, Mise H, Kakui K, Korita D, et al Role of leptin in pregnancy—a review. Placenta. 2002;23 Suppl A:S80–6. Epub 2002/04/30. doi: 10.1053/plac.2002.0814 . 11978063 10.1053/plac.2002.0814

[10] Benirschke K, Burton G, Baergen R. Pathology of the Human Placenta. 6th Edition ed. New York: Springer; 2012.

[11] Arizawa M. Clinical Placentology (Japanese). first ed. Kinpodo: Tokyo; 2013.

[12] Yamazaki K, Masaki N, Kohmura-Kobayashi Y, Yaguchi C, Hayasaka T, Itoh H, et al Decrease in Sphingomyelin (d18:1/16:0) in Stem Villi and Phosphatidylcholine (16:0/20:4) in Terminal Villi of Human Term Placentas with Pathohistological Maternal Malperfusion. PLoS One. 2015;10(11):e0142609 Epub 2015/11/17. doi: 10.1371/journal.pone.0142609 . 26569622 10.1371/journal.pone.0142609 PMC4646668

[13] Nakamura Y, Yaguchi C, Itoh H, Sakamoto R, Kimura T, Furuta N, et al Morphologic characteristics of the placental basal plate in in vitro fertilization pregnancies: a possible association with the amount of bleeding in delivery. Hum Pathol. 2015;46(8):1171–9. Epub 2015/06/11. doi: 10.1016/j.humpath.2015.04.007 . 26058728 10.1016/j.humpath.2015.04.007

[14] Jansson T, Powell TL. Role of the placenta in fetal programming: underlying mechanisms and potential interventional approaches. Clin Sci (Lond). 2007;113(1):1–13. doi: 10.1042/CS20060339 . 17536998 10.1042/CS20060339

[15] Khalife N, Glover V, Hartikainen AL, Taanila A, Ebeling H, Jarvelin MR, et al Placental size is associated with mental health in children and adolescents. PLoS One. 2012;7(7):e40534 doi: 10.1371/journal.pone.0040534 . 22792364 10.1371/journal.pone.0040534 PMC3392232

[16] Beebe LA, Cowan LD, Altshuler G. The epidemiology of placental features: associations with gestational age and neonatal outcome. Obstet Gynecol. 1996;87(5 Pt 1):771–8. . 8677084 10.1016/0029-7844(95)00483-1

[17] Chisholm KM, Folkins AK. Placental and Clinical Characteristics of Term Small-for-Gestational-Age Neonates: A Case-Control Study. Pediatr Dev Pathol. 2016;19(1):37–46. Epub 2015/09/15. doi: 10.2350/15-04-1621-OA.1 . 26368794 10.2350/15-04-1621-OA.1

[18] Elimian A, Verma U, Beneck D, Cipriano R, Visintainer P, Tejani N. Histologic chorioamnionitis, antenatal steroids, and perinatal outcomes. Obstet Gynecol. 2000;96(3):333–6. Epub 2000/08/29. . 10960621 10.1016/s0029-7844(00)00928-5

[19] Catov JM, Scifres CM, Caritis SN, Bertolet M, Larkin J, Parks WT. Neonatal outcomes following preterm birth classified according to placental features. Am J Obstet Gynecol. 2017;216(4):411.e1–e14. doi: 10.1016/j.ajog.2016.12.022 . 28065815 10.1016/j.ajog.2016.12.022

[20] Tsuchiya KJ, Matsumoto K, Suda S, Miyachi T, Itoh H, Kanayama N, et al Searching for very early precursors of autism spectrum disorders: the Hamamatsu Birth Cohort for Mothers and Children (HBC). J Dev Orig Health Dis. 2010;1(3):158–73. Epub 2010/06/01. doi: 10.1017/S2040174410000140 . 25141784 10.1017/S2040174410000140

[21] Takagai S, Tsuchiya KJ, Itoh H, Kanayama N, Mori N, Takei N. Cohort Profile: Hamamatsu Birth Cohort for Mothers and Children (HBC Study). Int J Epidemiol. 2016;45(2):333–42. Epub 2015/11/01. doi: 10.1093/ije/dyv290 . 26519951 10.1093/ije/dyv290

[22] Khong TY, Mooney EE, Ariel I, Balmus NC, Boyd TK, Brundler MA, et al Sampling and Definitions of Placental Lesions: Amsterdam Placental Workshop Group Consensus Statement. Arch Pathol Lab Med. 2016;140(7):698–713. doi: 10.5858/arpa.2015-0225-CC . 27223167 10.5858/arpa.2015-0225-CC

[23] Kraus F, Redline R, Gersell D, Nelson M, Dicke J. Placental Pathology (Atlas of Nontumor Pathology) Washington, DC: The American Registry of Pathology; 2004.

[24] Redline RW. Classification of placental lesions. Am J Obstet Gynecol. 2015;213(4 Suppl):S21–8. doi: 10.1016/j.ajog.2015.05.056 . 26428500 10.1016/j.ajog.2015.05.056

[25] Fogarty NM, Ferguson-Smith AC, Burton GJ. Syncytial knots (Tenney-Parker changes) in the human placenta: evidence of loss of transcriptional activity and oxidative damage. Am J Pathol. 2013;183(1):144–52. doi: 10.1016/j.ajpath.2013.03.016 . 23680657 10.1016/j.ajpath.2013.03.016

[26] Kim YM, Chaemsaithong P, Romero R, Shaman M, Kim CJ, Kim JS, et al Placental lesions associated with acute atherosis. J Matern Fetal Neonatal Med. 2014:1–9. doi: 10.3109/14767058.2014.960835 . 25183023 10.3109/14767058.2014.960835 PMC4416076

[27] Desa DJ. Intimal cushions in foetal placental veins. J Pathol. 1973;110:347–52.

[28] Baergen RN. Manual of Pathology of the Human Placenta. 2 ed. Springer 2011.

[29] Seidmann L, Suhan T, Kamyshanskiy Y, Nevmerzhitskaya A, Gerein V, Kirkpatrick CJ. CD15—a new marker of pathological villous immaturity of the term placenta. Placenta. 2014;35(11):925–31. doi: 10.1016/j.placenta.2014.07.018 . 25149387 10.1016/j.placenta.2014.07.018

[30] Conti N, Torricelli M, Voltolini C, Vannuccini S, Clifton VL, Bloise E, et al Term histologic chorioamnionitis: a heterogeneous condition. Eur J Obstet Gynecol Reprod Biol. 2015;188:34–8. Epub 2015/03/17. doi: 10.1016/j.ejogrb.2015.02.034 . 25770845 10.1016/j.ejogrb.2015.02.034

[31] Redline RW. Villitis of unknown etiology: noninfectious chronic villitis in the placenta. Hum Pathol. 2007;38(10):1439–46. doi: 10.1016/j.humpath.2007.05.025 . 17889674 10.1016/j.humpath.2007.05.025

[32] de Onis M. 4.1 The WHO Child Growth Standards. World Rev Nutr Diet. 2015;113:278–94. doi: 10.1159/000360352 . 25906897 10.1159/000360352

[33] Macara L, Kingdom JC, Kaufmann P, Kohnen G, Hair J, More IA, et al Structural analysis of placental terminal villi from growth-restricted pregnancies with abnormal umbilical artery Doppler waveforms. Placenta. 1996;17(1):37–48. . 8710812 10.1016/s0143-4004(05)80642-3

[34] Kingdom JC, Kaufmann P. Oxygen and placental villous development: origins of fetal hypoxia. Placenta. 1997;18(8):613–21; discussion 23–6. 9364596. 9364596 10.1016/s0143-4004(97)90000-x

[35] Redline RW. Placental inflammation. Semin Neonatol. 2004;9(4):265–74. doi: 10.1016/j.siny.2003.09.005 . 15251143 10.1016/j.siny.2003.09.005

[36] Kim CJ, Romero R, Chaemsaithong P, Chaiyasit N, Yoon BH, Kim YM. Acute chorioamnionitis and funisitis: definition, pathologic features, and clinical significance. Am J Obstet Gynecol. 2015;213(4 Suppl):S29–52. doi: 10.1016/j.ajog.2015.08.040 . 26428501 10.1016/j.ajog.2015.08.040 PMC4774647

[37] Williams MC, O’Brien WF, Nelson RN, Spellacy WN. Histologic chorioamnionitis is associated with fetal growth restriction in term and preterm infants. Am J Obstet Gynecol. 2000;183(5):1094–9. doi: 10.1067/mob.2000.108866 . 11084547 10.1067/mob.2000.108866

[38] Imura H. Life course health care and preemptive approach to non-communicable diseases. Proc Jpn Acad Ser B Phys Biol Sci. 2013;89(10):462–73. doi: 10.2183/pjab.89.462 . 24334510 10.2183/pjab.89.462 PMC3883454

[39] Agboola SO, Ball M, Kvedar JC, Jethwani K. The future of Connected Health in preventive medicine. QJM. 2013;106(9):791–4. doi: 10.1093/qjmed/hct088 . 23598385 10.1093/qjmed/hct088

[40] Hanson MA, Gluckman PD. Developmental origins of health and disease—Global public health implications. Best Pract Res Clin Obstet Gynaecol. 2014 doi: 10.1016/j.bpobgyn.2014.06.007 . 25225058 10.1016/j.bpobgyn.2014.06.007

[41] Gupta S, Venkatesh A, Ray S, Srivastava S. Challenges and prospects for biomarker research: a current perspective from the developing world. Biochim Biophys Acta. 2014;1844(5):899–908. doi: 10.1016/j.bbapap.2013.12.020 . 24412545 10.1016/j.bbapap.2013.12.020

[42] Gomez-Lopez N, Guilbert LJ, Olson DM. Invasion of the leukocytes into the fetal-maternal interface during pregnancy. J Leukoc Biol. 2010;88(4):625–33. doi: 10.1189/jlb.1209796 . 20519637 10.1189/jlb.1209796

