# Supplementary material for: Placental pathology predicts infantile physical development during first 18 months in Japanese population: Hamamatsu birth cohort for mothers and children (HBC Study)
Source: PLoS One. 2018 Apr 10;13(4):e0194988. doi: 10.1371/journal.pone.0194988 (PMC5892873; doi:10.1371/journal.pone.0194988)
Supplement: S1 File — Means and SDs of weight and the ponderal index (PI), stratified by the negative/positive of each placental pathological finding. (DOCX) [file pone.0194988.s001.docx]

**Supplemental Tables. Means and SDs of weight and the ponderal index (PI), stratified by the negative/positive of each placental pathological finding.**

| **A** |  |  | |  |  | |  | |  |
| --- | --- | --- | --- | --- | --- | --- | --- | --- | --- |
| Placental pathological finding of ‘Accelerated villous maturation’ | | | | | | | | | |
| month | | Accelerated villous maturation | | | | Accelerated villous maturation | | | P |
|  |  | negative | | | | positive | | |  |
|  |  | mean | Std. Dev | | | mean | | Std. Dev |  |
| Body weight (g) | **0** | **2871.3** | **519.1** | | | **2569.5** | | **586.8** | **<0.001** |
|  | **1** | **3927.4** | **642.4** | | | **3521.9** | | **716.1** | **<0.001** |
|  | **4** | **6338.8** | **795.2** | | | **5928.0** | | **724.7** | **<0.001** |
|  | 6 | 7107.4 | 760.6 | | | 6875.0 | | 799.4 | 0.035 |
|  | 10 | 8506.8 | 814.6 | | | 8297.7 | | 746.7 | 0.066 |
|  | 14 | 9426.7 | 883.8 | | | 9217.6 | | 812.3 | 0.090 |
|  | 18 | 10093.7 | 986.9 | | | 9896.2 | | 914.7 | 0.152 |
| PI (g/cm^3^) | 0 | 2.421 | 0.251 | | | 2.433 | | 0.326 | 0.743 |
|  | 1 | 2.653 | 0.224 | | | 2.582 | | 0.226 | 0.026 |
|  | 4 | 2.749 | 0.286 | | | 2.699 | | 0.326 | 0.236 |
|  | 6 | 2.640 | 0.245 | | | 2.670 | | 0.287 | 0.409 |
|  | 10 | 2.419 | 0.205 | | | 2.411 | | 0.183 | 0.778 |
|  | 14 | 2.206 | 0.143 | | | 2.208 | | 0.168 | 0.940 |
|  | 18 | 1.999 | 0.162 | | | 1.991 | | 0.131 | 0.705 |

0 months indicates birth weight. Significance was set at a p value of 0.003 as described in the Methods.

| **B** |  |  | |  |  |  | |  |
| --- | --- | --- | --- | --- | --- | --- | --- | --- |
| Placental pathological finding of ‘Decidual vasculopathy’ | | | | | | | | |
| month | | Decidual vasculopathy | | | Decidual vasculopathy | | | p |
|  |  | negative | | | positive | | |  |
|  |  | Mean | Std. Dev | | Mean | | Std. Dev |  |
| Body weight (g) | 0 | 2844.4 | 548.5 | | 2700.1 | | 550.5 | 0.044 |
|  | 1 | 4034.7 | 772.6 | | 3858.5 | | 709.0 | 0.108 |
|  | 4 | 6457.3 | 764.8 | | 6244.2 | | 772.3 | 0.060 |
|  | 6 | 7401.8 | 943.8 | | 7071.3 | | 848.2 | 0.075 |
|  | 10 | 8440.5 | 902.2 | | 8246.8 | | 839.8 | 0.137 |
|  | 14 | 8877.8 | 1051.2 | | 8681.6 | | 871.8 | 0.383 |
|  | 18 | 9955.9 | 1116.2 | | 9824.2 | | 950,7 | 0.404 |
| PI (g/cm^3^) | 0 | 2.431 | 0.287 | | 2.411 | | 0.244 | 0.567 |
|  | 1 | 2.713 | 0.249 | | 2.682 | | 0.249 | 0.386 |
|  | 4 | 2.731 | 0.267 | | 2.753 | | 0.257 | 0.576 |
|  | 6 | 2.584 | 0.274 | | 2.564 | | 0.227 | 0.714 |
|  | 10 | 2.402 | 0.182 | | 2.441 | | 0.202 | 0.170 |
|  | 14 | 2.163 | 0.173 | | 2.173 | | 0.210 | 0.803 |
|  | 18 | 2.027 | 0.174 | | 2.020 | | 0.164 | 0.807 |

0 months indicates birth weight. Significance was set at a p value of 0.003 as described in the Methods.

| **C** |  |  | |  |  |  |  |
| --- | --- | --- | --- | --- | --- | --- | --- |
| Placental pathological finding of ‘Thrombosis or Intramural fibrin deposition’ | | | | | | | |
| month | | Thrombosis or  Intramural fibrin deposition | | | Thrombosis or  Intramural fibrin deposition | | p |
|  |  | negative | | | positive | |  |
|  |  | Mean | | Std. Dev. | Mean | Std. Dev. |  |
| Body weight (g) | 0 | 2754.5 | 564.9 | | 2884.9 | 513.6 | 0.084 |
|  | 1 | 3942.5 | 753.8 | | 4035.4 | 755.5 | 0.480 |
|  | 4 | 6306.1 | 803.0 | | 6560.1 | 667.9 | 0.031 |
|  | 6 | 7204.1 | 1001.4 | | 7435.4 | 739.9 | 0.214 |
|  | 10 | 8309.2 | 970.3 | | 8500.3 | 637.4 | 0.158 |
|  | 14 | 8602.0 | 1078.8 | | 9153.2 | 696.6 | 0.013 |
|  | 18 | 9834.4 | 1131.0 | | 10083.7 | 845.7 | 0.132 |
| PI (g/cm^3^) | 0 | 2.426 | 0.256 | | 2.420 | 0.309 | 0.869 |
|  | 1 | 2.690 | 0.247 | | 2.734 | 0.200 | 0.250 |
|  | 4 | 2.735 | 0.262 | | 2.749 | 0.266 | 0.723 |
|  | 6 | 2.584 | 0.267 | | 2.566 | 0.243 | 0.740 |
|  | 10 | 2.417 | 0.182 | | 2.417 | 0.208 | 0.997 |
|  | 14 | 2.183 | 0.189 | | 2.137 | 0.184 | 0.310 |
|  | 18 | 2.025 | 0.171 | | 2.022 | 0.168 | 0.881 |

0 months indicates birth weight. Significance was set at a p value of 0.003 as described in the Methods.

| **D** |  |  |  |  | |  |  |
| --- | --- | --- | --- | --- | --- | --- | --- |
| Placental pathological finding of ‘Avascular villi’ | | | | | | | |
| month | | Avasular villi | | Avasular villi | | | p |
|  |  | negative | | positive | | |  |
|  |  | Mean | Std. Dev | Mean | | Std. Dev |  |
| Body weight (g) | 0 | 2785.9 | 559.4 | | 2855.5 | 492.5 | 0.543 |
|  | 1 | 3968.1 | 751.6 | | 4010.0 | 789.2 | 0.806 |
|  | 4 | 6378.4 | 779.8 | | 6410.5 | 726.6 | 0.852 |
|  | 6 | 7273.4 | 943.2 | | 7360.4 | 793.7 | 0.736 |
|  | 10 | 8361.1 | 891.5 | | 8432.6 | 824.0 | 0.721 |
|  | 14 | 8782.3 | 1010.3 | | 8976.2 | 827.9 | 0.563 |
|  | 18 | 9867.1 | 1064.9 | | 10286.1 | 941.4 | 0.102 |
| PI (g/cm^3^) | 0 | 2.421 | 0.267 | | 2.454 | 0.318 | 0.548 |
|  | 1 | 2.705 | 0.249 | | 2.677 | 0.245 | 0.617 |
|  | 4 | 2.746 | 0.264 | | 2.689 | 0.250 | 0.328 |
|  | 6 | 2.582 | 0.252 | | 2.552 | 0.295 | 0.680 |
|  | 10 | 2.417 | 0.191 | | 2.417 | 0.189 | 0.992 |
|  | 14 | 2.162 | 0.189 | | 2.205 | 0.181 | 0.521 |
|  | 18 | 2.020 | 0.169 | | 2.063 | 0.179 | 0.295 |

0 months indicates birth weight. Significance was set at a p value of 0.003 as described in the Methods.

| **E** |  |  | |  |  |  | |  |
| --- | --- | --- | --- | --- | --- | --- | --- | --- |
| Placental pathological finding of ‘Delayed villous maturation’ | | | | | | | | |
| month | | Delayed villous maturation | | | Delayed villous maturation | | | p |
|  |  | negative | | | positive | | |  |
|  |  | Mean | Std. Dev | | Mean | | Std. Dev |  |
| Body weight (g) | 0 | 2805.2 | 548.5 | | 2700.1 | | 550.5 | 0.466 |
|  | 1 | 4034.7 | 772.6 | | 3858.5 | | 709.0 | 0.108 |
|  | 4 | 6457.3 | 764.8 | | 6244.2 | | 772.3 | 0.060 |
|  | 6 | 7401.8 | 943.8 | | 7071.3 | | 848.2 | 0.075 |
|  | 10 | 8440.5 | 902.2 | | 8246.8 | | 839.8 | 0.137 |
|  | 14 | 8877.8 | 1051.2 | | 8681.6 | | 871.8 | 0.383 |
|  | 18 | 9955.9 | 1116.2 | | 9824.2 | | 950.7 | 0.404 |
| PI (g/cm^3^) | 0 | 2.431 | 0.287 | | 2.411 | | 0.244 | 0.567 |
|  | 1 | 2.713 | 0.249 | | 2.682 | | 0.249 | 0.386 |
|  | 4 | 2.731 | 0.267 | | 2.753 | | 0.257 | 0.576 |
|  | 6 | 2.584 | 0.274 | | 2.564 | | 0.227 | 0.714 |
|  | 10 | 2.402 | 0.182 | | 2.441 | | 0.202 | 0.170 |
|  | 14 | 2.163 | 0.173 | | 2.173 | | 0.210 | 0.803 |
|  | 18 | 2.027 | 0.174 | | 2.020 | | 0.164 | 0.807 |

0 months indicates birth weight. Significance was set at a p value of 0.003 as described in the Methods.

| **F** |  |  | |  |  |  | |  |
| --- | --- | --- | --- | --- | --- | --- | --- | --- |
| Placental pathological finding of ‘Maternal inflammatory response’ | | | | | | | | |
| month | | Maternal inflammatory response | | | Maternal inflammatory response | | | p |
|  |  | negative | | | positive | | |  |
|  |  | Mean | Std. Dev. | | Mean | | Std. Dev. |  |
| Body weight (g) | **0** | **2689.6** | **579.1** | | **2948.3** | | **471.4** | **<0.001** |
|  | **1** | **3680.9** | **705.1** | | **4034.5** | | **595.0** | **<0.001** |
|  | **4** | **6085.1** | **808.8** | | **6453.4** | | **727.9** | **<0.001** |
|  | **6** | **6876.9** | **720.9** | | **7303.0** | | **789.0** | **<0.001** |
|  | 10 | 8340.6 | 837.1 | | 8619.1 | | 716.5 | 0.006 |
|  | **14** | **9229.9** | **917.7** | | **9586.8** | | **744.4** | **0.001** |
|  | 18 | 9912.9 | 998.9 | | 10237.2 | | 896.8 | 0.008 |
| PI (g/cm^3^) | 0 | 2.423 | 0.300 | | 2.425 | | 0.225 | 0.967 |
|  | 1 | 2.617 | 0.236 | | 2.660 | | 0.208 | 0.143 |
|  | 4 | 2.736 | 0.294 | | 2.735 | | 0.303 | 0.979 |
|  | 6 | 2.632 | 0.253 | | 2.672 | | 0.261 | 0.215 |
|  | 10 | 2.427 | 0.187 | | 2.402 | | 0.215 | 0.317 |
|  | 14 | 2.204 | 0.160 | | 2.210 | | 0.133 | 0.740 |
|  | 18 | 1.992 | 0.155 | | 2.004 | | 0.153 | 0.518 |

0 months indicates birth weight. Significance was set at a p value of 0.003 as described in the Methods.

| **G** |  |  | |  |  |  | |  |
| --- | --- | --- | --- | --- | --- | --- | --- | --- |
| Placental pathological finding of ‘Fetal inflammatory response’ | | | | | | | | |
| month | | Fetal inflammatory response | | | Fetal inflammatory response | | | p |
|  |  | negative | | | positive | | |  |
|  |  | Mean | Std. Dev. | | Mean | | Std. Dev. |  |
| Body weight (g) | **0** | **2720.4** | **557.3** | | **3060.4** | | **445.4** | **<0.001** |
|  | **1** | **3726.8** | **696.5** | | **4173.7** | | **505.0** | **<0.001** |
|  | **4** | **6127.0** | **771.7** | | **6620.2** | | **774.3** | **<0.001** |
|  | **6** | **6945.6** | **706.4** | | **7421.5** | | **904.4** | **<0.001** |
|  | 10 | 8389.7 | 803.5 | | 8682.0 | | 756.8 | 0.016 |
|  | 14 | 9307.1 | 883.1 | | 9613.4 | | 775.9 | 0.020 |
|  | 18 | 9980.7 | 981.0 | | 10270.1 | | 904.5 | 0.049 |
| PI (g/cm^3^) | 0 | 2.414 | 0.287 | | 2.462 | | 0.206 | 0.237 |
|  | 1 | 2.631 | 0.238 | | 2.646 | | 0.174 | 0.661 |
|  | 4 | 2.731 | 0.292 | | 2.754 | | 0.316 | 0.606 |
|  | 6 | 2.646 | 0.246 | | 2.656 | | 0.293 | 0.795 |
|  | 10 | 2.429 | 0.185 | | 2.374 | | 0.239 | 0.071 |
|  | 14 | 2.210 | 0.156 | | 2.192 | | 0.124 | 0.430 |
|  | 18 | 1.988 | 0.150 | | 2.030 | | 0.164 | 0.068 |

0 months indicates birth weight. Significance was set at a p value of 0.003 as described in the Methods.

| **H** |  |  | |  |  |  | |  |
| --- | --- | --- | --- | --- | --- | --- | --- | --- |
| Placental pathological finding of ‘VUE’ | | | | | | | | |
| month | | VUE | | | VUE | | | p |
|  |  | negative | | | positive | | |  |
|  |  | Mean | Std. Dev | | Mean | | Std. Dev |  |
| Body weight (g) | 0 | 2792.4 | 555.6 | | 2795.7 | | 537.2 | 0.982 |
|  | 1 | 3820.4 | 695.6 | | 3796.9 | | 476.9 | 0.897 |
|  | 4 | 6237.8 | 812.0 | | 6141.9 | | 544.1 | 0.652 |
|  | 6 | 7061.2 | 781.1 | | 6802.4 | | 692.3 | 0.211 |
|  | 10 | 8470.5 | 806.9 | | 8107.8 | | 653.9 | 0.100 |
|  | 14 | 9382.3 | 880.2 | | 9183.4 | | 689.5 | 0.390 |
|  | 18 | 10057.7 | 981.3 | | 9771.3 | | 793.8 | 0.269 |
| PI (g/cm^3^) | 0 | 2.422 | 0.274 | | 2.448 | | 0.263 | 0.726 |
|  | 1 | 2.631 | 0.227 | | 2.665 | | 0.187 | 0.564 |
|  | 4 | 2.737 | 0.301 | | 2.711 | | 0.237 | 0.739 |
|  | 6 | 2.652 | 0.251 | | 2.568 | | 0.341 | 0.220 |
|  | 10 | 2.416 | 0.201 | | 2.427 | | 0.169 | 0.840 |
|  | 14 | 2.207 | 0.150 | | 2.202 | | 0.146 | 0.900 |
|  | 18 | 1.995 | 0.152 | | 2.028 | | 0.185 | 0.422 |

0 months indicates birth weight. Significance was set at a p value of 0.003 as described in the Methods.

| **I** |  |  | |  |  |  | |  |
| --- | --- | --- | --- | --- | --- | --- | --- | --- |
| Placental pathological finding of ‘VUE’ | | | | | | | | |
| month | | Deciduitis | | | Deciduitis | | | p |
|  |  | negative | | | positive | | |  |
|  |  | Mean | Std. Dev. | | Mean | | Std. Dev. |  |
| Body weight (g) | 0 | 2792.2 | 548.0 | | 2804.7 | | 648.4 | 0.935 |
|  | 1 | 3824.6 | 677.2 | | 3778.3 | | 829.0 | 0.806 |
|  | 4 | 6234.6 | 791.2 | | 6189.7 | | 921.6 | 0.838 |
|  | 6 | 7050.2 | 761.2 | | 6992.6 | | 1035.2 | 0.788 |
|  | 10 | 8458.7 | 805.1 | | 8341.2 | | 751.0 | 0.595 |
|  | 14 | 9369.7 | 866.3 | | 9418.4 | | 949.7 | 0.840 |
|  | 18 | 10054.4 | 965.2 | | 9833.0 | | 1080.9 | 0.408 |
| PI (g/cm^3^) | 0 | 2.423 | 0.273 | | 2.445 | | 0.258 | 0.771 |
|  | 1 | 2.641 | 0.225 | | 2.518 | | 0.223 | 0.048 |
|  | 4 | 2.740 | 0.301 | | 2.658 | | 0.202 | 0.314 |
|  | 6 | 2.654 | 0.257 | | 2.539 | | 0.229 | 0.103 |
|  | 10 | 2.422 | 0.201 | | 2.332 | | 0.141 | 0.099 |
|  | 14 | 2.211 | 0.147 | | 2.127 | | 0.181 | 0.040 |
|  | 18 | 2.002 | 0.153 | | 1.901 | | 0.147 | 0.017 |

0 months indicates birth weight. Significance was set at a p value of 0.003 as described in the Methods.

| J |  |  |  |  |  |  |
| --- | --- | --- | --- | --- | --- | --- |
| Placental pathological finding of ‘Maternal vascular malperfusion’ | | | | | | |
| month | | Maternal vascular malperfusion | | Maternal vascular malperfusion | | p |
|  |  | negative | | positive | |  |
|  |  | Mean | Std. Dev. | Mean | Std. Dev. |  |
| Body weight (g) | 0 | 2885.8 | 530.7 | 2686.0 | 560.0 | 0.003 |
|  | **1** | **4122.9** | **731.4** | **3795.3** | **744.9** | **0.002** |
|  | 4 | 6499.8 | 795.2 | 6244.2 | 724.8 | 0.018 |
|  | 6 | 7419.1 | 990.2 | 7130.5 | 816.1 | 0.105 |
|  | 10 | 8492.0 | 947.1 | 8235.7 | 790.3 | 0.040 |
|  | 14 | 8868.2 | 1114.0 | 8279.6 | 844.9 | 0.554 |
|  | 18 | 9982.4 | 1152.3 | 9824.4 | 941.0 | 0.298 |
| PI (g/cm^3^) | 0 | 2.417 | 0.256 | 2.433 | 0.290 | 0.617 |
|  | 1 | 2.727 | 0.244 | 2.673 | 0.252 | 0.116 |
|  | 4 | 2.730 | 0.255 | 2.750 | 0.273 | 0.574 |
|  | 6 | 2.605 | 0.283 | 2.545 | 0.223 | 0.252 |
|  | 10 | 2.410 | 0.189 | 2.424 | 0.192 | 0.618 |
|  | 14 | 2.166 | 0.179 | 2.167 | 0.199 | 0.982 |
|  | 18 | 2.033 | 0.179 | 2.014 | 0.159 | 0.432 |

0 months indicates birth weight. Significance was set at a p value of 0.003 as described in the Methods.

| **K** |  |  | |  |  |  | |  |
| --- | --- | --- | --- | --- | --- | --- | --- | --- |
| Placental pathological finding of ‘Fetal vascular malperfusion’ | | | | | | | | |
| month | | Fetal vascular malperfusion | | | Fetal vascular malperfusion | | | p |
|  |  | negative | | | positive | | |  |
|  |  | Mean | | Std. Dev. | Mean | Std. Dev. | |  |
| Body weight (g) | 0 | 2748.5 | 575.3 | | 2881.7 | | 495.1 | 0.068 |
|  | 1 | 3929.9 | 765.7 | | 4047.7 | | 731.4 | 0.281 |
|  | 4 | 6287.5 | 803.0 | | 6567.1 | | 676.6 | 0.014 |
|  | 6 | 7157.7 | 1001.1 | | 7478.6 | | 755.3 | 0.076 |
|  | 10 | 8283.8 | 956.7 | | 8524.9 | | 708.0 | 0.066 |
|  | 14 | 8600.0 | 1081.3 | | 9107.5 | | 749.0 | 0.199 |
|  | 18 | 9789.2 | 1113.0 | | 10153.8 | | 895.8 | 0.023 |
| PI (g/cm^3^) | 0 | 2.431 | 0.253 | | 2.410 | | 0.306 | 0.552 |
|  | 1 | 2.686 | 0.022 | | 2.731 | | 0.244 | 0.208 |
|  | 4 | 2.738 | 0.267 | | 2.743 | | 0.257 | 0.898 |
|  | 6 | 2.586 | 0.274 | | 2.565 | | 0.234 | 0.692 |
|  | 10 | 2.414 | 0.184 | | 2.421 | | 0.202 | 0.799 |
|  | 14 | 2.187 | 0.191 | | 2.133 | | 0.178 | 0.229 |
|  | 18 | 2.024 | 0.172 | | 2.024 | | 0.170 | 0.997 |

0 months indicates birth weight. Significance was set at a p value of 0.003 as described in the Methods.
